# Identification of Pharmacological Modulators of HMGB1-Induced Inflammatory Response by Cell-Based Screening

**DOI:** 10.1371/journal.pone.0065994

**Published:** 2013-06-14

**Authors:** Domokos Gerö, Petra Szoleczky, Katalin Módis, John P. Pribis, Yousef Al-Abed, Huan Yang, Sangeeta Chevan, Timothy R. Billiar, Kevin J. Tracey, Csaba Szabo

**Affiliations:** 1 Department of Anesthesiology, University of Texas Medical Branch, Galveston, Texas, United States of America; 2 Department of Surgery, University of Pittsburgh, Pittsburgh, Pennsylvania, United States of America; 3 Laboratory of Biomedical Science, Feinstein Institute for Medical Research, Manhasset, New York, United States of America; University of Pittsburgh, United States of America

## Abstract

High mobility group box 1 (HMGB1), a highly conserved, ubiquitous protein, is released into the circulation during sterile inflammation (e.g. arthritis, trauma) and circulatory shock. It participates in the pathogenesis of delayed inflammatory responses and organ dysfunction. While several molecules have been identified that modulate the release of HMGB1, less attention has been paid to identify pharmacological inhibitors of the downstream inflammatory processes elicited by HMGB1 (C23-C45 disulfide C106 thiol form). In the current study, a cell-based medium-throughput screening of a 5000+ compound focused library of clinical drugs and drug-like compounds was performed in murine RAW264.7 macrophages, in order to identify modulators of HMGB1-induced tumor-necrosis factor alpha (TNFα) production. Clinically used drugs that suppressed HMGB1-induced TNFα production included glucocorticoids, beta agonists, and the anti-HIV compound indinavir. A re-screen of the NIH clinical compound library identified beta-agonists and various intracellular cAMP enhancers as compounds that potentiate the inhibitory effect of glucocorticoids on HMGB1-induced TNFα production. The molecular pathways involved in this synergistic anti-inflammatory effect are related, at least in part, to inhibition of TNFα mRNA synthesis via a synergistic suppression of ERK/IκB activation. Inhibition of TNFα production by prednisolone+salbutamol pretreatment was also confirmed in vivo in mice subjected to HMGB1 injection; this effect was more pronounced than the effect of either of the agents administered separately. The current study unveils several drug-like modulators of HMGB1-mediated inflammatory responses and offers pharmacological directions for the therapeutic suppression of inflammatory responses in HMGB1-dependent diseases.

## Introduction

High-mobility group box 1 protein (HMGB1) was initially considered a nuclear protein regulating gene transcription. However, data emerging over the last decade identified its separate role as a pro-inflammatory cytokine that is released actively and passively from cells during inflammation and injury [1]–[3]. According to a current classification, the immune response can be regulated by endogenous danger signals (damage-associated molecular patterns; DAMPs; alarmins) as well as exogenous pathogen-associated molecular patterns (PAMPs). In this context, HMGB1 has been identified as a *bona fide* DAMP (i.e. a mediator released during sterile inflammatory processes), as well as a mediator released during PAMP-associated inflammatory events (e.g. sepsis and septic shock), which participates in the pathogenesis of the delayed inflammatory response, organ injury and contributes to disease mortality [1]–[3].

Significant work has focused on the molecular mechanisms of HMGB1 release and on the therapeutic neutralization of HMGB1, either by antibodies, or by inhibiting its binding to its receptors RAGE and TLR4 [1]–[7]. Several compounds have been identified that attenuate the *release of HMGB1*, including glucocorticoids, chloroquine, gold salts, nicotinic receptor agonists, ethyl pyruvate and inhibitors of poly(ADP-ribose) polymerase [3], [8]–[11]. However, the inflammatory cellular responses *downstream from HMGB1* are less understood, and no systematic survey has been conducted to characterize these pathways or to identify their pharmacological modulators. One determinant of the bioactivity of extracellular HMGB1 is based on the redox status of its three conserved thiol groups. The all thiol confirmation has been show to facilitate the binding of CXCL12 to CXCR4 and thus exhibit chemokine-like properties [12]. The C23-C45 disulfide C106 thiol conformation binds to the CD14/MD2/TLR4 receptor complex [13], [14] and demonstrates cytokine-like properties. Using a cell-based medium-throughput screening approach, the goal of the current study was to identify drug-like compounds that down-regulate the cytokine-like activity of HMGB1-induced inflammatory processes in murine macrophages *in vitro*.

## Methods

### Materials and Reagents

A comprehensive screening set of 5,546 compounds was gathered comprising the NIH Clinical Collection (446 phase I–III trial compounds) from BioFocus (South San Francisco, CA), the FDA Approved Library (640 FDA approved bioactive compounds) from Enzo Life Sciences (Farmingdale, NY), the Prestwick Chemical Library (1200 marketed drugs in Europe) from Prestwick Chemical (Washington, DC), the US Drug Collection (1040 clinical trial stage USP drugs), the International Drug Collection (240 compounds marketed in Europe or Asia but not in the US) and Killer Plates (160 toxic substances) from MicroSource Discovery Systems (Gaylordsville, CT), the LOPAC1280 (1280 various biologically active compounds) from Sigma-Aldrich, (Saint Louis, MO) and the Natural Products (640 natural compounds and derivatives) from TimTec LLC (Newark, DE). The compounds were dissolved at 10 mM in dimethyl-sulfoxide (DMSO) and dilutions were made either in DMSO or in phosphate-buffered saline (PBS, pH 7.4) to obtain 0.5% final DMSO concentration. HMGB1 (C23-C45 disulfide C106 thiol form) was prepared as previously described [15] and diluted in OptiMEM I medium (Invitrogen, Carlsbad, CA). Unless specified otherwise, all other reagents were purchased from Sigma-Aldrich Co. (St. Louis, MO).

### Cell Culture

RAW 264.7 murine macrophages were obtained from the American Type Culture Collection (ATCC, Manassas, VA) and maintained in Dulbecco’s modified Eagle’s medium (DMEM) (Hyclone, Logan, UT) containing 4.5 g/l glucose supplemented with 10% fetal bovine serum (FBS, PAA Laboratories Inc, Westborough, MA), 100 IU/ml penicillin and 100 µg/ml streptomycin (Invitrogen, Carlsbad, CA) at 37°C in 5% CO_2_ atmosphere. Prior to HMGB1 stimulation the culture medium was replaced with OptiMEM I reduced serum medium (Invitrogen, Carlsbad, CA).

### Screening Assay

RAW 264.7 cells (100 000/well) were plated into 96-well tissue culture plates and cultured overnight. Culture medium was replaced with OptiMEM prior to adding compounds. Test compounds were supplied at 10 mM in dimethyl sulfoxide (DMSO) and were diluted in DMSO and in phosphate buffer saline (PBS) to reach 3 µM final concentration (and 0.5% DMSO) in the culture medium. The Natural Products Library was screened at 1 µg/ml final concentration. Compounds were administered in 1/20 volume 1 hour prior to HMGB1 treatment. In the combined screen the cells received additional dexamethasone (3 µM) treatment. HMGB1 was added at 5 µg/ml final concentration in 1/10 volume and the cells were incubated for 18 hours at 37°C in 5% CO_2_ atmosphere. Supernatant was collected to measure TNFα secretion and LDH release.

### Viability (MTT Assay) and LDH Release Measurements

The MTT assay and LDH activity measurements were performed as previously described [16]. Briefly, the cells were incubated in medium containing 0.5 mg·mL^−1^,3-(4,5-dimethyl-2-thiazolyl)-2,5-diphenyl-2H-tetrazolium bromide (MTT, Calbiochem, EMD BioSciences, San Diego, CA) for 1 hour at 37°C at 5% CO_2_ atmosphere. The converted formazan dye was dissolved in isopropanol and the absorbance was measured at 570 nm. Serial dilution of the cells was used to calculate the count of viable cells. Viability values are shown as percent values relative to vehicle treated controls. LDH release was measured by mixing cell culture supernatant (30 µl) with 100 µl LDH assay reagent containing 110 mM lactic acid, 1350 mM nicotinamide adenine dinucleotide (NAD^+^), 290 mM *N*-methylphenazonium methyl sulfate (PMS), 685 mM 2-(4-Iodophenyl)-3-(4-nitrophenyl)-5-phenyl-2*H*-tetrazolium chloride (INT) and 200 mM Tris (pH 8.2). The changes in absorbance were read kinetically at 492 nm for 15 min (kinetic LDH assay). LDH activity values are shown as Vmax (mOD/min).

### TNFα ELISA

Supernatant was diluted 10 times in PBS containing 1% bovine serum albumin (BSA) and the TNFα levels were determined with a commercially available ELISA kit (R&D Systems, Minneapolis, MN) on a robotic system comprising of a plate washer (EL406, Biotek, Winooski, VT), a dispenser (MicroFlo, Biotek, Winooski, VT), a pipetting station (Precision, Biotek, Winooski, VT), an incubator (Cytomat 2C, Thermo Electron Corporation, Asheville, NC) and plate reader (Synergy 2, Biotek, Winooski, VT) connected with a robotic arm (Twister II, Caliper Life Sciences Inc, Hopkinton, MA).

### RNA Isolation, Gene Expression Measurements

Total RNA was isolated from RAW 264.7 cells exposed to HMGB1 or vehicle for 1.5 or 6 hours using a commercial RNA purification kit (SV total RNA isolation kit, Promega, Madison, WI). 2 µg RNA was reverse transcribed using the High Capacity cDNA Archive kit (Applied Biosystems, Foster City, CA) as previously described [17]. 1 µg RNA was used according to the manufacturer’s protocol for gene expression measurements using the Toll-like receptor signaling pathway real-time PCR array (PAMM-0018ZD, SA Biosciences, Frederick, MD) on CFX96 thermocycler (Biorad, Hercules, CA) and analyzed with the tool provided by SA Biosciences. A full list of the genes investigated is deposited in Table S1. Taqman assay for TNFα was performed using a commercial assay (TNFα assay ID: Mm00443260_g1, Life Technologies, Carlsbad, CA) using GAPDH (VIC/MGB Probe, Applied Biosystems, Foster City, CA) control as normalizer.

### Western Blotting

Cells were lysed in denaturing loading buffer (20 mM Tris, 2% SDS, 10% glycerol, 6 M urea, 100 µg/ml bromophenol blue, 200 mM ß-mercaptoethanol) freshly supplemented with 2 mM sodium vanadate, 100 mM sodium fluoride, 20 mM beta-glycerophosphate and protease inhibitors (Complete Mini EDTA-free, Roche Applied Science, Indianapolis, IN). Lysates were sonicated, boiled and resolved on 4–12% NuPage Bis-Tris acrylamide gels (Invitrogen, Carlsbad, CA), then transferred to nitrocellulose. Membranes were blocked in 10% non-fat dried milk and probed overnight with phospho-ERK1/2, (Cell Signaling, Boston, MA), phospho-p38 or phospho-IκB antibodies (Santa Cruz Biotechnology Inc, Santa Cruz, CA). After incubation with peroxidase conjugates the blots were detected on a CCD-camera based detection system (GBox, Syngene USA, Frederick, MD) with enhanced chemiluminescent substrate. To normalize signals, membranes were stripped in 62.5 mM Tris, 2% SDS, 100 mM ß-mercaptoethanol at 60°C for 30 min, blocked and re-probed with antibodies against ERK1/2, p38 and IκB. The signals were quantitated using Genetools analysis software (Syngene USA, Frederick, MD).

### Pharmacological Modulation of HMGB1-induced TNFα Production *in vivo*

This study was carried out in strict accordance with the recommendations in the Guide for the Care and Use of Laboratory Animals of the National Institutes of Health. The protocol was approved by the Committee on the Ethics of Animal Experiments of the University of Texas Medical Branch, Galveston (Permit Number: 1110054). The procedures were performed humanely with minimal suffering. 6–7 week-old Balb/c male mice (The Jackson Laboratory) were pretreated subcutaneously with 20 mg/kg prednisolone, 10 mg/kg salbutamol, the combination of prednisolone and salbutamol (doses as above), or the glucocorticoid receptor blocker mifepristone (30 mg/kg) or the β-receptor antagonist propranolol (10 mg/kg) or vehicle for 3 hours. Mice were injected intraperitoneally (i.p.) with 0.5 mg/mouse HMGB1 and animals were sacrificed 8 hours later. Serum levels of TNFα were measured by ELISA (as above).

#### Statistical analysis

Data are shown as means ± SEM. One-way ANOVA was applied for statistical analysis and for the determination of significance, the Tukey’s post-hoc test was used. A p value of <0.05 was considered statistically significant. All statistical calculations were performed using Graphpad Prism 4 analysis software. Experiments were performed at least 3 times on different days.

## Results

### HMGB1 Induces Inflammatory Mediator Production and Cytotoxicity in RAW 264.7 Macrophages

**Figure 1:**
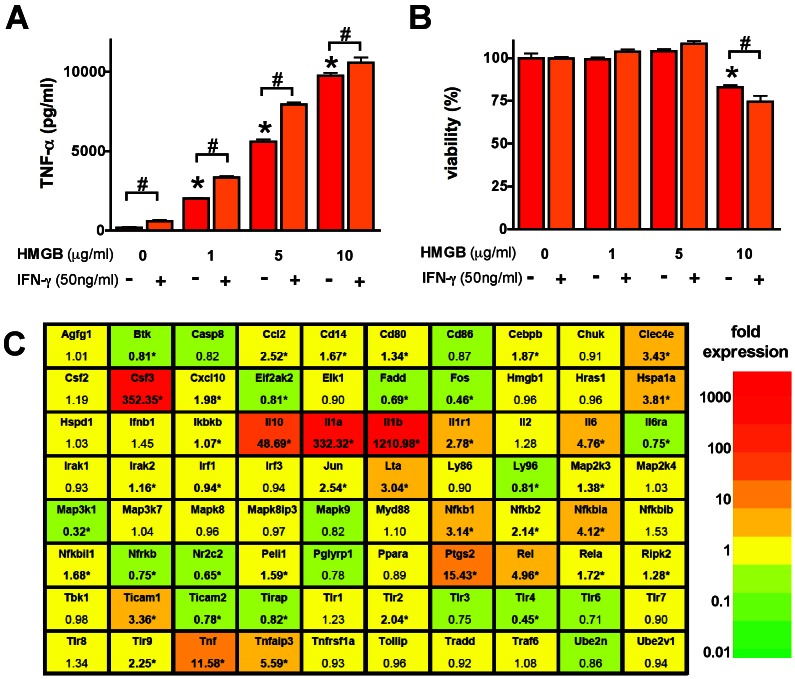
HMGB1 induces an inflammatory response in RAW 264.7 macrophages. **A–B:** RAW 264.7 cells were treated with the indicated amount of HMGB1 and IFN-γ for 18 hours and the TNFα secretion was measured in the supernatant. The viability of the cells was measured by the MTT assay. (*p<0.05 compared to vehicle treated cells, ^#^p<0.05 IFN-γ treated group compared to the respective HMGB1-treated group) **C:** RAW 264.7 cells were treated with HMGB1 (5 µg/ml) for 1.5 hours and the expression of TLR-associated genes was analyzed with TLR signaling pathways array. The gene symbols and the average fold-expression values are shown compared to vehicle-treated cells in the color-scale, according to the their relative expression. (*p<0.05 compared to vehicle-treated cells.).

**Figure 2:**
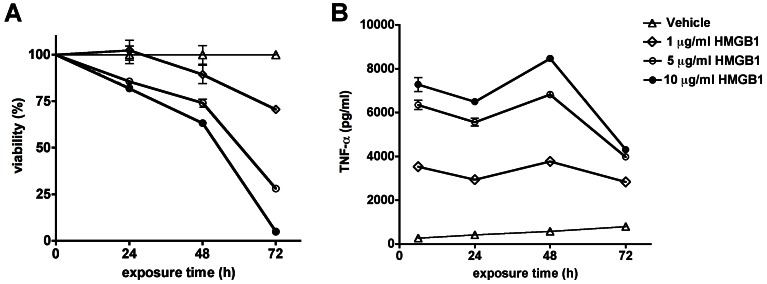
Concentration- and time-dependence of the HMGB1-induced inflammatory response and reduction in cell viability in RAW 264.7 macrophages. RAW 264.7 cells were treated with the indicated amount of HMGB1 for 24, 48 or 72 hours. **A:** Cell viability was measured with the MTT assay and **B:** TNFα secretion was measured in the supernatant.

**Figure 3:**
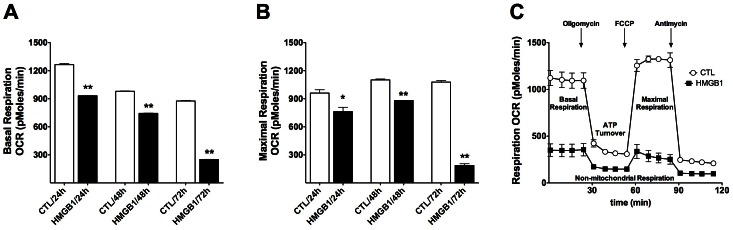
Time-dependence of the HMGB1-induced suppression of cellular bioenergetics in RAW 264.7 macrophages. RAW 264.7 cells were exposed to HMGB1 (5 µg/ml) for 24, 48 or 72 hours. Cellular bioenergetic parameters were measured with Seahorse extracellular fluid analysis. **A:** Time-dependent decrease in basal cellular respiration (Oxygen Consumption Rate, OCR). (**p<0.01 compared to vehicle treated cells) **B:** Time-dependent decrease in maximal cellular respiration. (*p<0.05 and **p<0.01 compared to vehicle treated cells). **C:** Representative tracing comparing cellular respiration (Oxygen Consumption Rate) in response to sequential administration of pharmacological modulators of cell metabolism in vehicle-treated cells or cells treated with HMGB1 for 72 hours. Basal Respiration, Calculated ATP Turnover, Proton Leak and Maximal Respiration areas are indicated and demonstrate a marked suppression of cellular bioenergetic parameters.

**Figure 4:**
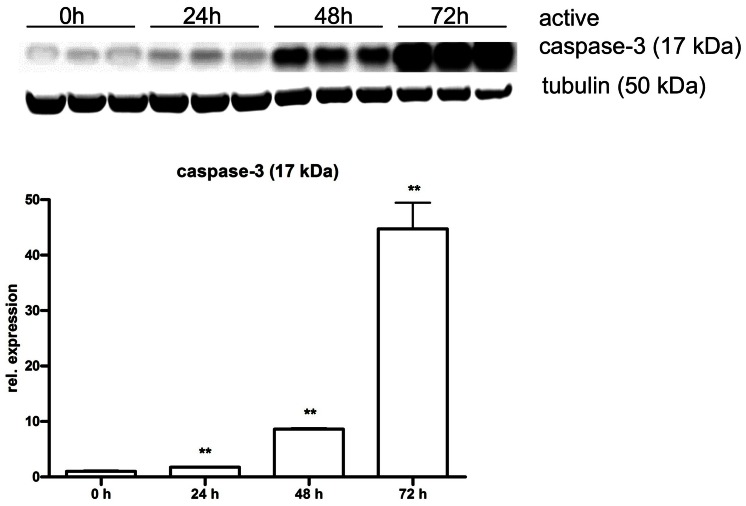
HMGB1 induces time-dependent caspase activation in RAW 264.7 macrophages. RAW 264.7 cells were exposed to HMGB1 (5 µg/ml) for 24, 48 or 72 hours. Activated Caspase-3 was detected in cell extracts by Western blotting. Tubulin was used for loading control. The graph shows relative Caspase-3 activation values, normalized to tubulin. (**p<0.01 shows significant caspase activation compared to vehicle-treated cells).

HMGB1 (1–10 µg/ml) induced concentration-dependent tumor necrosis factor α (TNFα) secretion by RAW 264.7 cells, an effect, which was potentiated by IFN-γ (Fig. 1A). HMGB1 also reduced cell viability (Fig. 1B); this cytotoxic response became more pronounced at later time points (48 h, 72 h) (Fig. 2) and was associated with a suppression of mitochondrial function (Fig. 3) and caspase activation (Fig. 4). In addition to TNFα, HMGB1 also upregulated multiple pro-inflammatory cytokine (IL1a, IL1b, IL6, TNFβ) and chemokine (Ccl2, MCP-1, Cxcl10) genes, as well as the anti-inflammatory cytokine IL10 (Fig. 1C). The HMGB1-mediated responses were also associated with an upregulation of nuclear factor κB (NF-κB) (Fig. 1C). Moreover, HMGB1 induced a down-regulation of TLR4 and MD2 and upregulation of TLR2, TLR9 and TLR adaptor molecule 1 (Ticam1) (Fig. 1C). Thus, the form of HMGB1 used for the screen exhibited the expected cytokine like properties of C23-C45 disulfide C106 thiol HMGB1.

### Identification of Inhibitors of HMGB1-induced TNFα Production by Cell-based Screening

**Figure 5:**
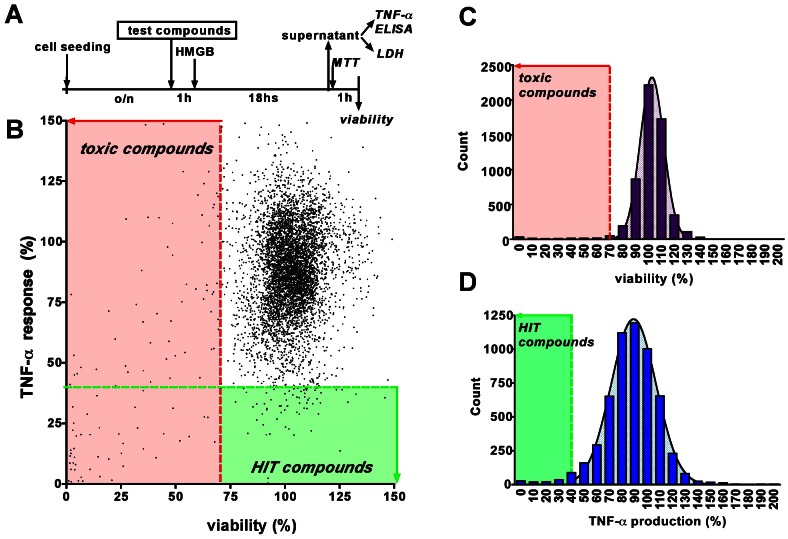
Screening for compounds that reduce the HMGB1-induced pro-inflammatory response. **A:** Timeline of the cell-based screening: RAW 264.7 cells were pre-treated with test compounds and exposed to HMGB1 for 18 hours. TNFα production was measured from the supernatant and the viability of the cells was measured by the MTT assay. **B:** Dot graph showing the individual TNFα/viability results of the tested 5,646 compounds. TNFα responses are shown as % values of the HMGB1-induced TNFα production. Values lower than MEAN-2SD are shown in red (viability) and green (TNFα response) boxes to denote “toxic” and “Hit” compounds. **C–D:** Distribution of viability (C) and TNFα response (D) data with superimposed Gaussian distribution curves fitted to the data points.

Cell-based screening of a focused library of over 5,000 clinical drugs, natural products and pharmacologically active compounds identified ∼2% of the compounds, which suppressed TNFα production, without adversely affecting cell viability (Table 1; Fig. 5). Conversely, a limited number of compounds induced a significant enhancement of HMGB1-mediated TNFα response (Table 2). A full list of the primary screen data is deposited in Table S2.

**Table 1:** List of hit compounds identified in the primary screen.

Name	Library	Biological activity	TNFα production (% of HMGB1 stimulation)	viability (%)
piperlongumine	Natural Products	anti-inflammatory, antioxidant	0	94
indapamide	ENZO FDA	sulphonamide diuretic	0	92
parthenolide	Prestwick	MAP kinase inhibitor anti-inflammatory	2	92
stattic	LOPAC	STAT3 activation inhibitor	14	125
parthenolide	LOPAC	MAP kinase inhibitor anti-inflammatory	20	104
U0126	LOPAC	inhibitor of MEK1 and MEK2 (MAP kinase kinase)	20	92
rubescensin A	Microsource Killer	antibacterial, antineoplastic	21	93
2′,4′-dihydroxyflavone	Natural Products	flavone	21	83
ethylnorepinephrin	Microsource US Drug	adrenergic agonist, bronchodilator	22	112
parthenolide	Natural Products	MAP kinase inhibitor anti-inflammatory	23	102
dexamethasone	Prestwick	glucocorticoid steroid	23	100
betamethasone	Prestwick	glucocorticoid steroid	24	90
metaproterenol	Prestwick	beta-adrenergic agonist, bronchodilator	26	87
budesonide	Prestwick	glucocorticoid steroid	27	96
dexamethasone	Microsource US Drug	glucocorticoid steroid	29	107
hydrocortisone base	Prestwick	glucocorticoid steroid	29	115
tolnaftate	Prestwick	antifungal	29	90
fludrocortisone	Prestwick	mineralocorticoid, glucocorticoid	30	98
dexamethasone	Microsource US Drug	glucocorticoid steroid	30	106
fenoterol hydrobromide	Prestwick	beta-adrenergic agonist, bronchodilatator	31	100
tyloxapol	Microsource US Drug	polymeric nonionic detergent	31	92
triamcinolone	Prestwick	glucocorticoid steroid	31	92
6-alpha-methylprednisolone	Prestwick	glucocorticoid steroid	31	110
flumethasone	Microsource US Drug	glucocorticoid steroid	31	109
ST057244	Natural Products		32	85
isoalantolactone	Natural Products	sesquiterpene lactone	32	101
ritodrine	Prestwick	beta_2_ adrenergic agonist, tocolytic	32	98
imipenem	Prestwick	antibiotic	32	101
clenbuterol	Prestwick	beta-adrenergic agonist, bronchodilatator	32	98
prenylamine	Prestwick	Ca^++^ channel blocker, vasodilator	33	87
hydrocortisone	Natural Products	glucocorticoid steroid	33	113
lidocaine	Prestwick	Na^+^ channel blocker, local anesthetic	33	91
prednisolone	Prestwick	glucocorticoid steroid	33	107
flurandrenolide	Microsource US Drug	glucocorticoid steroid	33	116
prednisolone	Microsource US Drug	glucocorticoid steroid	34	110
bethamethasone	Microsource US Drug	glucocorticoid steroid	34	129
fluticasone	Prestwick	glucocorticoid steroid	34	78
clenbuterol	Microsource Intl Drug	beta2-adrenergic agonist, bronchodilatator	35	111
ibudilast	LOPAC	phosphodiesterase IV inhibitor	35	101
Bay 11-7085	LOPAC	IκB phosphorylation inhibitor, inhibitor of NF-κB	35	94
fluocinolone	Microsource US Drug	glucocorticoid steroid	35	115
tulobuterol	Microsource Intl Drug	beta-adrenergic agonist, bronchodilatator	35	111
clobetasol propionate	Prestwick	glucocorticoid steroid	36	146
flurandrenolide	Prestwick	glucocorticoid steroid	36	107
prednisolone	Natural Products	glucocorticoid steroid	36	106
flunisolide	Microsource US Drug	glucocorticoid steroid	36	109
dexamethasone	Natural Products	glucocorticoid steroid	37	104
flumethasone	Prestwick	glucocorticoid steroid	37	133
albuterol	Microsource US Drug	beta-adrenergic agonist, bronchodilatator	37	100
hydrocortisone	Natural Products	glucocorticoid steroid	37	109
flumethasone	Microsource US Drug	glucocorticoid steroid	38	117
nordihydroguaiaretic acid	Natural Products	from creosote bush, Larrea divaricata	38	92
2,6-dimethoxyquinone	Microsource Killer	antibacterial, mutagen	38	82
dichlorisone	Microsource Int Drug	glucocorticoid steroid	38	107
salbutamol	Prestwick	beta-adrenergic agonist, bronchodilatator	38	102
bethamethasone	Microsource US Drug	glucocorticoid steroid	39	114
hydrocortisone	LOPAC	glucocorticoid steroid	39	110
triamcinolone	ENZO FDA	glucocorticoid steroid	39	109
desoxymetasone	Microsource US Drug	glucocorticoid steroid	39	126
mometasone	Prestwick	glucocorticoid steroid	39	124
Bay 11-7082	LOPAC	IκB phosphorylation inhibitor, inhibitor of NF-κB	39	95
prednisolone	Microsource US Drug	glucocorticoid steroid	39	110
fluorometholone	Prestwick	glucocorticoid steroid	39	104
3,7,4′-trihydroxyflavone	Natural Products	flavone	39	81
budesonide	Microsource US Drug	glucocorticoid steroid	39	110
halomethasone	NIH Clinical Collection	glucocorticoid steroid	39	106
fluorometholone	Microsource US Drug	glucocorticoid steroid	40	114
triamcinolone	Microsource US Drug	glucocorticoid steroid	40	115
ST009819	Natural Products	levoglucosenone derivative	40	101
prednisolone	ENZO FDA	glucocorticoid steroid	40	124
hydrocortisone	Microsource US Drug	glucocorticoid steroid	40	115
fluticasone	NIH Clinical Collection	glucocorticoid steroid	40	93
rimexolone	Prestwick	glucocorticoid steroid	40	96
isoproterenol	Prestwick	adrenergic agonist, bronchodilatator	40	109
methylprednisolone	Microsource US Drug	glucocorticoid steroid	41	115
methylprednisolone	ENZO FDA	glucocorticoid steroid	41	95
metaproterenol	Microsource US Drug	adrenergic agonist, bronchodilatator	41	104
karanjin	Natural Products	from Pongamia glabra, Leguminosae	41	100
salmeterol	ENZO FDA	beta_2_-adrenergic agonist, bronchodilator	41	111
betamethasone	ENZO FDA	glucocorticoid steroid	41	101
clobetasol	Microsource US Drug	glucocorticoid steroid	41	107
budesonide	LOPAC	glucocorticoid steroid	41	115
isotretinon	Microsource US Drug	antiacne, antineoplastic	41	115
benzyl isothiocyanate	Microsource Killer	antineoplastic, antibacterial, antifungal	41	89
quinacrine	Microsource Killer	anthelmintic, antimalarial, intercalating agent	42	77
flunisolide	Prestwick	glucocorticoid steroid	42	113
ellipticine	LOPAC	cytochrome P_450_ (CYP1A1) and DNA topoisomerase II inhibitor	42	95
terbutaline hemisulfate	Prestwick	beta_2_-adrenergic agonist, bronchodilator	42	96
alclometazone	Microsource US Drug	glucocorticoid steroid	42	112
methylprednisolone	Microsource US Drug	glucocorticoid steroid	42	113
2′,3′-dihydroxyflavone	Natural Products	flavone	42	88
(+)-dehydroabietylamine	Natural Products	ingredient of rosin amine, from Rosin Gum	42	90
triamcinolone	Microsource US Drug	glucocorticoid steroid	43	113
betamethasone	Microsource US Drug	glucocorticoid steroid	43	112
isoflupredone	Prestwick	glucocorticoid steroid	43	99
alclometasone	Prestwick	glucocorticoid steroid	43	147
p-aminobenzoate	Microsource US Drug	vitamin B_x_	43	118
dexamethasone	ENZO FDA	glucocorticoid steroid	43	113
indinavir	NIH Clinical Collection	HIV protease inhibitor	43	96
ethacrynic acid	Microsource US Drug	diuretic	43	96
beclomethasone	Microsource US Drug	glucocorticoid steroid	43	112
amcinonide	Microsource US Drug	glucocorticoid steroid	43	109
MNS	LOPAC	Src and Syk kinase inhibitor	43	93
sulfasalazine	Prestwick	prostaglandin 15-hydroxydehydrogenase inhibitor	44	97
betamethasone	Microsource US Drug	glucocorticoid steroid	44	119
fludrocortisone	Microsource US Drug	mineralocorticoid, glucocorticoid	44	115
5,3′-dihydroxyflavone	Natural Products	flavone derivative	44	99
methyl cholate	Natural Products		44	97
desonide	Microsource US Drug	glucocorticoid steroid	44	115
tolazoline	Prestwick	alpha adrenergic antagonist, vasodilator	44	87
vincristine	ENZO FDA	antineoplastic, microtubular polymerization inhibitor	44	94
ethacrynic acid	Prestwick	diuretic	45	98
levonordefrin	Prestwick	adrenergic agonist, vasoconstrictor	45	116
isofluprednone	Microsource US Drug	glucocorticoid steroid	45	123
4′-hydroxy-6-methoxyflavone	Natural Products	flavone	45	80
fluocinolone	ENZO FDA	glucocorticoid steroid	45	106
alprostadil	Prestwick	vasodilator, prostaglandin receptor agonist	45	82
maprotiline	Prestwick	antidepressant, noradrenaline uptake inhibitor	45	99
dobutamine	Prestwick	beta_1_-adrenergic agonist, bronchodilator	45	92
betamethasone	Microsource US Drug	glucocorticoid steroid	45	122
bromperidol	Prestwick	antipsychotic, dopamine antagonist	45	85

Non-toxic compounds that reduced the HMGB1-induced TNFα production by 2 standard deviation values are listed in order of potency, according to their inhibitory potency for TNFα secretion. The source library of the compounds, their known biological activity and the respective viability values are shown. Viability was measured by the MTT assay. (Abbreviations: MAP kinase: Mitogen-activated protein kinase, U0126∶1,4-diamino-2,3-dicyano-1,4-bis(2-aminophenylthio)butadiene, MEK: mitogen-activated protein kinase kinase, STAT3: Signal transducer and activator of transcription 3, ST057244∶1-[(2E)-3-(3,4,5-trimethoxyphenyl)prop-2-enoyl]piperidin-2-one, Bay 11-7085: (2*E*)-3-[[4-(1,1-dimethylethyl)pheny?l]sulfonyl]-2-propenenitrile, Bay 11-7082∶3- [(4- methylphenyl)sulfonyl]- (2E)- propenenitrile, ST009819: (2R,3R,13R,14R)-3-(phenylcarbonyl)-17,19-dioxa-4-azapentacyclo[14.2.1.0<2,14>. 0<4,13>.0<7,12>]nonadeca-5,7(12),8,10-tetraen-15-one, MNS: 3,4-methylenedioxy-β-nitrostyrene, IkB: inhibitor of nuclear factor κB kinase, NF-κB: nuclear factor κB, HIV: human immunodeficiency virus, Src: sarcoma tyrosine kinase, Syk: Spleen tyrosine kinase).

**Table 2:** Compounds that enhance the HMGB1-induced TNFα production of RAW264.7 cells.

Name	Library	Biological activity	TNFα production (% of HMGB1 stimulation)	viability (%)
beta-escin	Prestwick	an increaser of calcium permeability, venous insufficiency drug	351	101
thapsigargin	LOPAC	sarco-endoplasmic reticulum Ca^2+^-ATPase inhibitor	336	13
niclosamide	Microsource Killer	anthelmintic, uncouples oxidative phosphorylation	335	67
wortmannin	LOPAC	phosphatidylinositol 3-kinase inhibitor	330	93
calcimycin	LOPAC	Ca^2+^ ionophore	327	1
gossypol	LOPAC	proapoptotic, binds calmodulin, PKC inhibitor, anti-HIV	321	88
niclosamide	LOPAC	anthelmintic, uncouples oxidative phosphorylation	302	39
tyrphostin A9	LOPAC	PDGF tyrosine kinase receptor inhibitor	289	51
rottlerin	LOPAC	mitochondrial uncoupler	261	99
5-azacytidine	Prestwick	antineoplastic, pyrimidine antimetabolite	260	57
5-azacytidine	Microsource US Drug	antineoplastic, pyrimidine antimetabolite	257	84
vinblastine	Microsource US Drug	antineoplastic, spindle poison	256	86
imiquimod	Prestwick	immunomodulator, activator of Toll-Like Receptor 7	243	84
5-azacytidine	Microsource Killer	antineoplastic, pyrimidine antimetabolite	241	59
niclosamide	Prestwick	anthelmintic, uncouples oxidative phosphorylation	239	58
vincristine	Microsource US Drug	antineoplastic, microtubular agent	211	87
ivermectin	ENZO FDA	antiparasitic	206	86
gossypol	Microsource Killer	proapoptotic, binds calmodulin, PKC inhibitor, anti-HIV	204	112
rottlerin	Natural Products	mitochondrial uncoupler	196	77
pararosaniline	Microsource US Drug	anthelmintic, antischistosomal	195	89
podophyllotoxin	Natural Products	antineoplastic, microtubular agent	188	78
colchicine	Microsource US Drug	antineoplastic, microtubular agent	187	79
cantharidic acid	LOPAC	protein phosphatase 1 and 2A inhibitor	177	37
podophyllotoxin	Microsource US Drug	antineoplastic, microtubular agent	171	84
fluvastatin	ENZO FDA	statin, HMG-CoA reductase inhibitor	168	127
tyrphostin AG 879	LOPAC	tyrosine kinase inhibitor with potent effects on TrkA	165	65
itavastatin	NIH Clin. Collection	statin, HMG-CoA reductase inhibitor	163	105
simvastatin	Prestwick	statin, HMG-CoA reductase inhibitor	162	106
fluvastatin	Prestwick	statin, HMG-CoA reductase inhibitor	162	111
8-azaguanine	Prestwick	antineoplastic, purine antimetabolite	159	74
methiazole	Prestwick	antiparasitic	159	77
N-oleoyldopamine	LOPAC	endogenous vanilloid, weak cannabinoid receptor ligand	158	24
oxaliplatin	ENZO FDA	antineoplastic DNA crosslinker	157	94
hexachlorophene	Microsource Killer	disinfectant, topical anti-infective, anti-bacterial agent	156	99
cerivastatin	NIH Clin. Collection	statin, HMG-CoA reductase inhibitor	156	100
colchicine	Microsource Killer	antineoplastic, microtubular agent	155	77
parbendazole	Prestwick	anthelmintic, microtubular agent	155	80
5-azacytidine	LOPAC	antineoplastic, pyrimidine antimetabolite	155	61
mevastatin	LOPAC	statin, HMG-CoA reductase inhibitor	153	110
tridihexethyl	Prestwick	anticholinergic antispasmodic	153	96
cerivastatin	ENZO FDA	statin, HMG-CoA reductase inhibitor	153	121
nordihydroguaiaretic acid	LOPAC	antioxidant from Larrea divaricata	153	113
norcantharidin	LOPAC	inhibitor of the serine/threonine protein phosphatase 2A	151	108
tannic acid	Microsource Killer	nonspecific enzyme/receptor blocker	150	99

Compounds augmenting the HMGB1-induced TNFα production by 2 standard deviation values are listed are listed in order of potency, according to their enhancing effect on TNFαα secretion. The source library of the compounds, their known biological activity and the respective viability values are shown. Viability was measured by the MTT assay. (Abbreviations: PKC: protein kinase C, HIV: human immunodeficiency virus, PDGF: platelet-derived growth factor, HMG-CoA: 3-hydroxy-3-methylglutaryl-coenzyme A, TrkA: TRK1-transforming tyrosine kinase protein).

More than 50% of the hit compounds that inhibited TNFα production were glucocorticoids (Fig. 5; Table 1). Beta-adrenergic agonists represented the second-most common class. The activity of the hit compounds was next confirmed at 3 and 10 µM. Since glucocorticoids and beta agonists showed a clear class action, only a subset of these compounds was retested. Apart from glucocorticoids and beta agonists, the highest inhibitory activity was detected for the NF-κB inhibitors Bay 11-7085 and parthenolide, and the antioxidant piperlongumine. Increasing the concentration of the compounds to 10 µM did not produce more pronounced inhibitory responses, but approximately 15% of the hit compounds became slightly cytotoxic at this concentration (Table 3).

**Table 3:** List of confirmed hit compounds.

Compound	Biological activity	Source Library	Primary screen		Hit confirmation	
			TNFα response(% of HMGB1 stimulation)	viability (%)	TNFα response (% of HMGB1 stimulation)	
					3 µM	10 µM
Parthenolide	NF-κB inhibitor	Prestwick	2	92	21±2	0±0*
Bay 11-7085	NF-κB inhibitor	LOPAC	35	94	25±1	2±1*
Ethylnorepinephrine	beta adrenergic agonist	US Drug	22	112	31±0	26±1
Halomethasone	glucocorticoid	NIH Clinical	39	106	36±2	34±1
Salbutamol	beta adrenergic agonist	Prestwick	38	102	37±1	37±6
Dexamethasone	glucocorticoid	Prestwick	23	100	39±3	30±2
Budesonide	glucocorticoid	LOPAC	41	115	41±3	38±1
Indinavir	HIV protease inhibitor	NIH Clinical	43	96	42±3	42±3
Ethacrynic acid	diuretic	US Drug	43	96	44±0	3±1*
Fluticasone	glucocorticoid	NIH Clinical	40	93	44±4	43±1*
Hydrocortisone	glucocorticoid	LOPAC	39	110	45±1	45±3
Metaproterenol	beta adrenergic agonist	Prestwick	26	87	48±3	50±4
Fenoterol	beta adrenergic agonist	Prestwick	31	100	51±1	56±1
Ritodrine	beta_2_ adrenergic agonist	Prestwick	32	98	51±4	48±6
Terbutaline	beta_2_ adrenergic agonist	Prestwick	42	96	51±2	50±3
Isoflupredone	glucocorticoid	Prestwick	43	99	52±5	51±2
Clenbuterol	beta adrenergic agonist	Prestwick	32	98	52±4	44±5
MNS	Src/Syk kinase inhibitor	LOPAC	43	93	54±2	9±1*
Ethacrynic acid	diuretic	Prestwick	45	98	59±5	39±2*
Levonordefrin	adrenergic agonist	Prestwick	45	116	64±1	56±8
PABA potassium salt	vitamin Bx	US Drug	43	118	65±1	59±1
Isoproterenol	adrenergic agonist	Prestwick	40	109	67±2	58±5
Prenylamine	calcium channel blocker	Prestwick	33	87	69±4	59±4
Tyloxapol	surfactant	US Drug	31	92	78±0	57±2
Isotretinoin	retinoid	US Drug	41	115	79±2	60±5
Piperlongumine	antioxidant in peppers	Nat. Prod.	0	94	80±1	39±3*
Lidocaine	local anesthetic	Prestwick	33	91	not tested	

Hit compounds of the primary screen were retested in replicates at 3 and 10 µM against HMGB1 and LPS and compounds are shown that decreased the HMGB**-**induced TNFα production by at least 40% in the hit confirmation experiments. (The majority of the glucocorticoids were not retested during the hit confirmation studies, since all glucocorticoids showed similar activity, confirming their class action.) TNFα production and viability values are shown for the primary screen and the TNFα production is shown for the hit confirmation experiments (Mean±SD). Compounds that reduced cell viability by at least 25% are labeled with an asterisk. (Abbreviations: Bay 11-7085: (2*E*)-3-[[4-(1,1-dimethylethyl)phenyl]sulfonyl]-2-propenenitrile, MNS: 3,4-methylenedioxy-β-nitrostyrene, PABA potassium salt: para-aminobenzoic acid potassium salt, NF-κB: nuclear factor κB, HIV: human immunodeficiency virus, Src: sarcoma tyrosine kinase, Syk: Spleen tyrosine kinase).

### Identification of Pharmacological Potentiators of Glucocorticoids by Cell-based Screening

**Figure 6:**
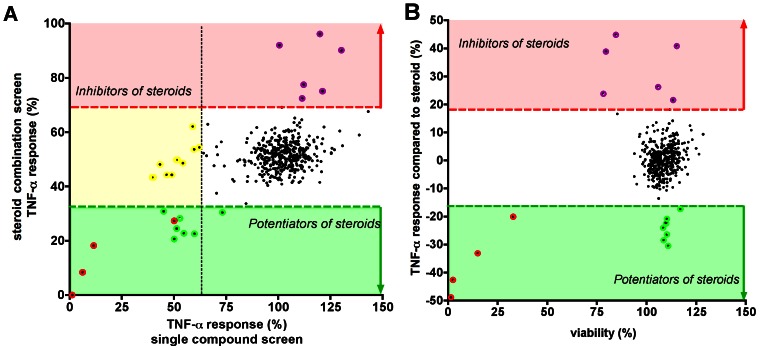
Combined screening to identify pharmacological potentiators of dexamethasone-mediated inhibition of the HMGB1-induced pro-inflammatory response. RAW 264.7 cells were pre-treated with dexamethasone (3 µM) in combination with test compounds and exposed to HMGB1 for 18 hours. TNFα production was measured from the supernatant and the viability of the cells was measured by the MTT assay. **A:** TNFα responses measured in the combination screen are plotted versus the TNFα production values measured in the single compound screen. TNFα production values higher than MEAN+2SD are shown in red (“steroid inhibitors”) and values lower than MEAN+2SD in green boxes (“potentiators of steroids) for the combination screen. Red dots denote the toxic compounds, green the steroid potentiators and purple those that increase the TNFα production. Compounds that inhibited the HMGB-induced TNFα production in the single compound screen, but failed to potentiate the action of steroids are shown in yellow. **B:** TNFα responses relative to the activity of dexamethasone are plotted versus the viability values. Red and green boxes indicate the upper and lower 2 SD limits.

We hypothesized that synergistic drug combinations may be more effective than single agents in controlling HMGB1-induced inflammatory responses. To identify compounds that potentiate the effect of glucocorticoids, a follow-up screen of the NIH Clinical Collection compound library was conducted in the presence of dexamethasone (3 µM). The screen identified beta2 agonists (salbutamol, salmeterol), the phosphodiesterase (PDE) inhibitor rolipram and as prostaglandin E1 as synergistic enhancers of the glucocorticoid's effect (Fig. 6, Table 4). In addition, the dopamine receptor antagonist SCH 23390 (*R*)-(+)-7-chloro-8-hydroxy-3-methyl-1-phenyl-2,3,4,5-tetrahydro-1*H*-3-benzazepine HCl), the structurally related benzodiazepine lorazepam, the antioxidant ebselen and the δ1 opioid receptor agonist SB 205607 decreased TNFα production in the presence of the glucocorticoid. As expected, the glucocorticoid receptor antagonist mifepristone attenuated the effect of dexamethasone (Table 4). A few drugs (e.g. cerivastatin, vindesine, vinorelbine) increased TNFα production in the presence dexamethasone (Table 4); this effect was related to the fact that, according to the results of the primary screen, these compounds, on their own, increase HMGB1-induced TNFα secretion (Table 1).

**Table 4:** Compounds of interest identified by screening of the NIH Library in the presence of 3 µM dexamethasone on HMGB1-induced TNFα production.

Compound	Biological activity	Single compound screen		Steroid combination screen		
		TNFα response (% of HMGB1 stimulation)	Viability (%)	TNFα response (% of HMGB1 stimulation)		Viability (%)
**Steroid action enhancers (potentiators)**						
salmeterol	beta_2_ adrenergic agonist	50	102		21	111
rolipram	phosphodiesterase inhibitor	60	86		23	109
prostaglandin E1	prostaglandin	55	97		23	109
salbutamol sulfate	beta_2_ adrenergic agonist	51	91		25	110
lorazepam	benzodiazepine	50	95		27	108
SB 205607	δ_1_ opioid receptor agonist	53	102		29	110
R(+)-SCH-23390 HCl	D_1_ dopamine receptor antagonist	73	96		30	110
ebselen	anti-inflammatory antioxidant	85	103		34	117
**Hit compounds in single-compound screen that do not enhance steroid action (non-potentiators)**						
fluticasone propionate	glucocorticoid steroid	40	93		43	108
halometasone monohydrate	glucocorticoid steroid	39	106		44	107
tadalafil	PDE5 inhibitor	49	107		44	108
tropisetron HCl	serotonin 5-HT_3_ antagonist	46	99		44	110
indinavir sulfate	HIV protease inhibitor	43	96		48	105
beclomethasone	glucocorticoid steroid	54	108		49	106
zaleplon	GABA A α_1_ agonist hypnotic	52	101		50	109
desoximetasone	glucocorticoid steroid	52	108		50	111
pergolide mesylate salt	dopamine receptor agonist	60	99		54	110
loteprednol etabonate	glucocorticoid steroid	59	104		62	108
**Compounds that counteract dexamethasone (steroid inhibitors)**						
cerivastatin Na	statin	>150	100		83	126
rutin	platelet aggregation inhibitor	102	97		69	112
ritonavir	HIV protease inhibitor	112	98		73	113
vinorelbine bitartarate	antineoplastic	112	78		78	106
vindesine sulfate	mitotic inhibitor	120	69		96	84
mifepristone	glucocorticoid receptor antagonist	101	101		92	115
**Toxic compounds**						
dactinomycin	antibiotics	1	1		0	2
triptolide	NF-κB inhibitor	0	2		0	1
homoharringtonine	60-S ribosome inhibitor	0	1		1	1
idarubicin HCl	anti-leukemic drug	6	2		9	2
epirubicin HCl	anthracycline drug	12	2		18	15
doxorubicin HCl	anthracycline drug	45	4		31	32
topotecan HCl	topoisomerase inhibitor	94	49		45	94
indarubicin	antineoplastic	100	72		54	94
diphenylcyclopropenone	local immune response inducer	114	71		56	100
artesunate	anti-malaria compound	121	74		75	78
vincristine sulfate	mitotic inhibitor	130	72		90	80

Compounds of interest are shown with their respective TNFα response and viability values attained in the single compound and combined screens. Drugs that reduced the TNFα response compared to the action of dexamethasone are classified as potentiators. Drugs that decreased the TNFα response by themselves, but showed negligible increase in their activity in combination with dexamethasone are listed as non-potentiators. Compounds that resulted in higher TNFα secretion (>MEAN+2SD) are listed as steroid inhibitors. Compounds that reduced the viability by more than 2 SD (<75% viability) are listed as toxic compounds.

### Glucocorticoid/beta-adrenergic Agonist Synergy: Mechanism of Action

**Figure 7:**
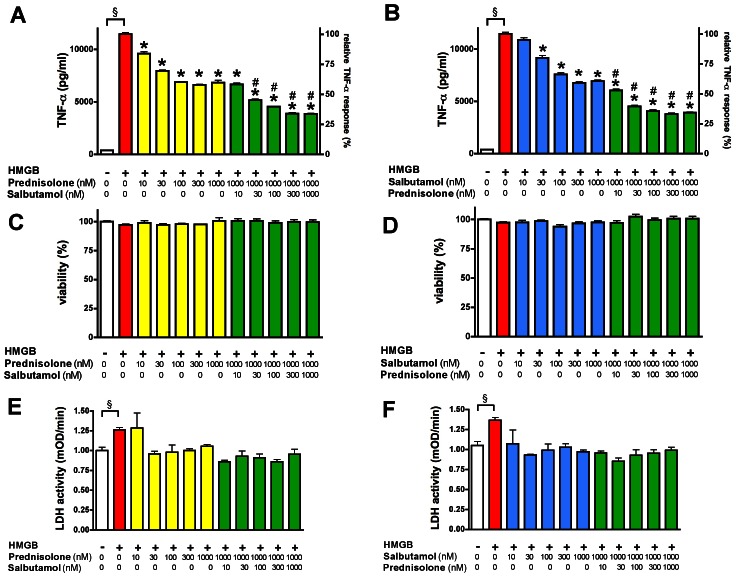
Prednisolone and salbutamol synergistically suppress HMGB1-induced TNFα secretion. RAW 264.7 cells were pretreated with prednisolone and salbutamol at the indicated concentrations and exposed to HMGB1 (5 µg/ml) for 18 hours. TNFα secretion (**A, B**) and LDH release (**E, F**) were measured in the supernatant. Cell viability (**C, D**) was measured by the MTT assay. (^§^p<0.05 HMGB1-treated group compared to vehicle treated control, *p<0.05 compared to HMGB1 group, ^#^p<0.05 compared to the respective first compound treatment).

Using prednisolone (a prototypical glucocorticoid) and salbutamol (a prototypical beta 2 adrenergic agonist), follow-up experiments were designed to further characterize the pharmacological properties and underlying mechanisms of the glucocorticoid/beta-adrenergic synergy. Both prednisolone and salbutamol, on their own, decreased the HMGB1-induced TNFα production in the low nanomolar concentration range: they reached their maximum effect at around 100–300 nM, exhibiting a 50% inhibition of TNFα production (Fig. 7). Salbutamol (1 µM), in combination with prednisolone, significantly reduced TNFα production already at 10 nM (compared to salbutamol alone); at 100 nM prednisolone the combination reached its full potential (approximately 70% inhibition). Likewise, the combination of prednisolone (1 µM) with 30 nM salbutamol significantly reduced the HMGB1-induced TNFα response (compared to prednisolone alone) and with 300 nM salbutamol the combination reached its full potential (approximately 70% inhibition) (Fig. 7).

**Figure 8:**
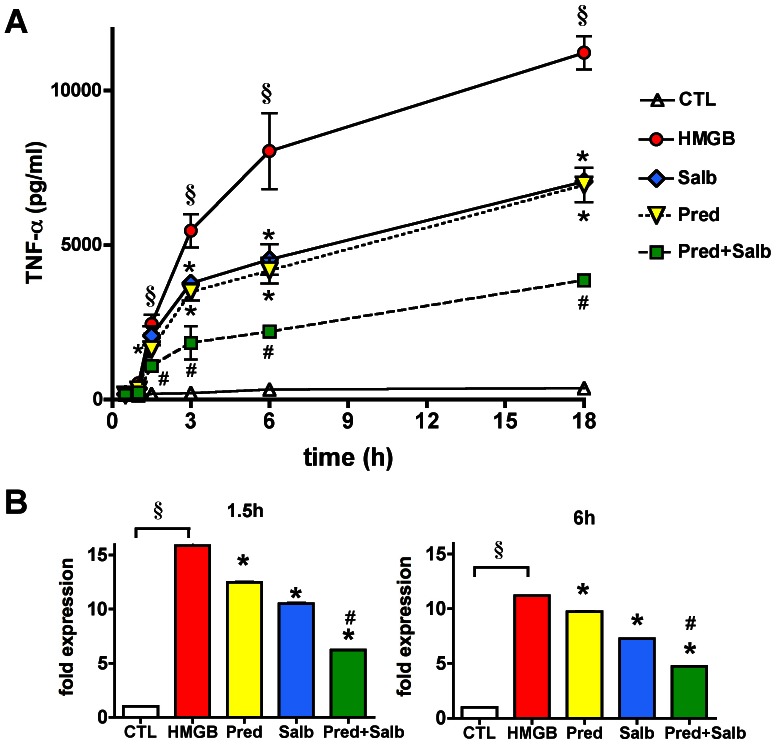
Prednisolone and salbutamol inhibit the HMGB-induced TNFα production. RAW 264.7 cells were pretreated with prednisolone (1 µM) and salbutamol (1 µM) and then exposed to HMGB1 (5 µg/ml) for various time up to 18 hours. **A**: TNFα secretion measured in the supernatant is plotted versus exposure length. (MEAN±SD values are shown) **B**: TNFα mRNA expression, normalized to glyceraldehyde 3-phosphate dehydrogenase (GAPDH), is shown as fold expression values of vehicle treated cells. (CTL: vehicle treated control, HMGB: cells exposed to HMGB1, Pred: cells pretreated with prednisolone and exposed to HMGB1, Salb: cells pretreated with salbutamol and exposed to HMGB1, Pred+Salb: cells pretreated with both prednisolone and salbutamol and exposed to HMGB1. ^§^p<0.05 HMGB1-treated group compared to vehicle treated control, *p<0.05 compared to HMGB1 group, ^#^p<0.05 compared to single compound treatment).

**Figure 9:**
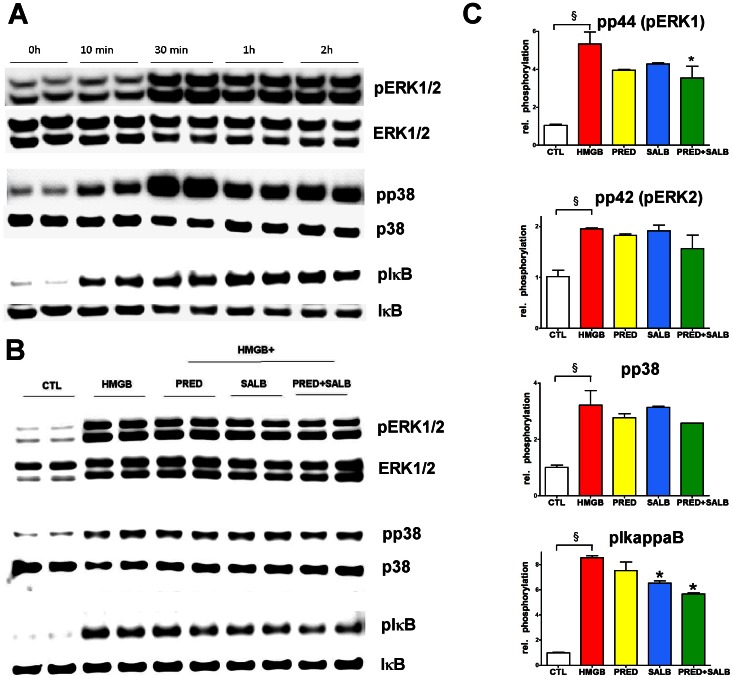
MAPK activation and IκB phosphorylation in response to HMGB1 are ameliorated in synergy by prednisolone and salbutamol. **A:** RAW 264.7 cells were exposed to HMGB1 (5 µg/ml) for the indicated length and the phosphorylation of ERK1/2, p38 and IκB was detected. **B:** RAW 264.7 cells pretreated with prednisolone (1 µM) and salbutamol (1 µM) were exposed to HMGB1 (5 µg/ml) for 30 min (ERK1/2, p38) or 1 hour (IκB) and the activation was detected as phospho-p44/42 MAPK (Erk1/2) (Thr202/Tyr204), phospho-p38 (Thr 180) or phospho-IκB-α (Ser 32/36). **C:** Bar graph shows the phosphorylation signal normalized to the total amount of the respective protein. (CTL: vehicle treated control, HMGB: cells exposed to HMGB1, Pred: cells pretreated with prednisolone and exposed to HMGB1, Salb: cells pretreated with salbutamol and exposed to HMGB1, Pred+Salb: cells pretreated with both prednisolone and salbutamol and exposed to HMGB1. ^§^p<0.05 HMGB1-treated group compared to vehicle treated control, *p<0.05 compared to HMGB1 group).

HMGB1-induced TNFαα secretion was associated with a rapid-onset and marked increase in TNFα mRNA (Fig. 8). Prednisolone and salbutamol each decreased the TNFα mRNA level by 50%; combination of the two compounds synergistically inhibited the transcription of TNFα mRNA (Fig. 8). We next tested whether the early inhibition of TNFα production involves upstream signaling events such as mitogen-activated protein kinase (MAPK) activation and IκB phosphorylation. HMGB1 induced an early and sustained activation of the extracellular signal-regulated kinases 1/2 (ERK1/2, p44 and p42) and p38 and of IκB phosphorylation (Fig. 9). The combination of salbutamol and prednisolone resulted in a partial, but statistically significant inhibition of ERK1 phosphorylation and IκB phosphorylation (Fig. 9). These data indicate the regulation of HMGB1-mediated cellular signaling by the glucocorticoid/beta-agonist combination has an upstream regulatory component.

**Figure 10:**
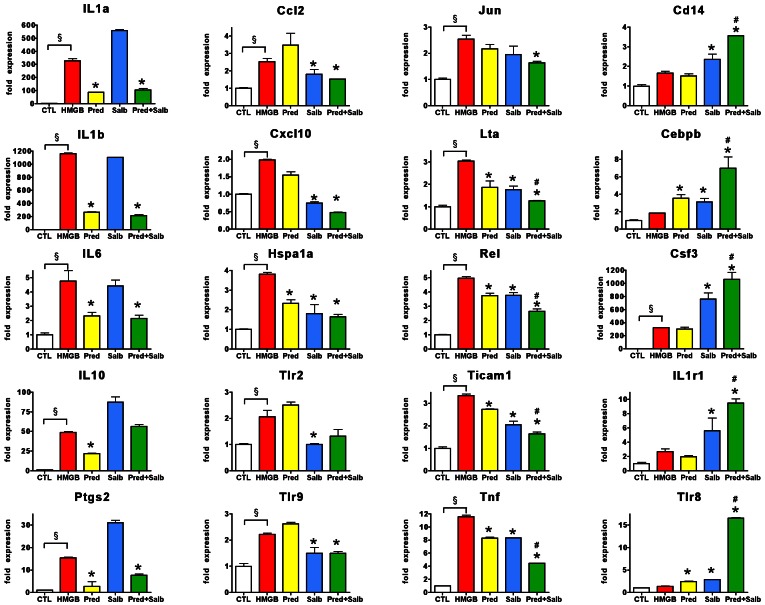
The interaction of prednisolone and salbutamol in the inhibition of HMGB-induced gene expression. RAW 264.7 cells pretreated with prednisolone (1 µM) and salbutamol (1 µM) were exposed to HMGB1 (5 µg/ml) for 1.5 hours and the expression of TLR-associated genes was analyzed with TLR signaling pathways array. Gene expression normalized to control genes (GAPDH, actin, B2m, Gusb, Hsp90ab1) is shown as fold expression values of vehicle treated cells. (CTL: vehicle treated control, HMGB: cells exposed to HMGB1, Pred: cells pretreated with prednisolone and exposed to HMGB1, Salb: cells pretreated with salbutamol and exposed to HMGB1, Pred+Salb: cells pretreated with both prednisolone and salbutamol and exposed to HMGB1. ^§^p<0.05 HMGB1-treated group compared to vehicle treated control, *p<0.05 compared to HMGB1 group, ^#^p<0.05 compared to single compound treatment).

To further characterize the effect of the glucocorticoid/beta-agonist combination on HMGB1-induced gene transcription, a TLR signaling pathway array was next employed. The responses could be characterized by four distinct expression patterns: **a)** prednisolone, but not salbutamol inhibiting gene expression, **b)** salbutamol, but not prednisolone inhibiting gene expression, **c)** the two compounds synergistically blocking gene expression and **d)** the two compounds synergistically enhancing gene expression (Fig. 10). The genes which were mostly inhibited by steroids included the interleukins (IL1a, Il1b, IL6, IL10) and Ptgs2 (COX-2); the inhibition exerted by the beta-2 agonist was dominant in case of the chemokines Ccl2 and Cxcl10 and TLR2 and TLR9; synergistic inhibition by the glucocorticoid and the beta-agonist was confirmed for TNFα, as well as demonstrated for lymphotoxin (Lta) and the TLR adaptor Ticam1 (Fig. 10). Unexpectedly, in a few instances, the steroid and the beta2 agonist led to a synergistic *enhancement*, as seen with Csf3 (GCSF), CD14, CCAAT/enhancer-binding protein beta (Cebpb), interleukin 1 receptor alpha (IL1R1) and TLR8 (Fig. 10).

**Figure 11:**
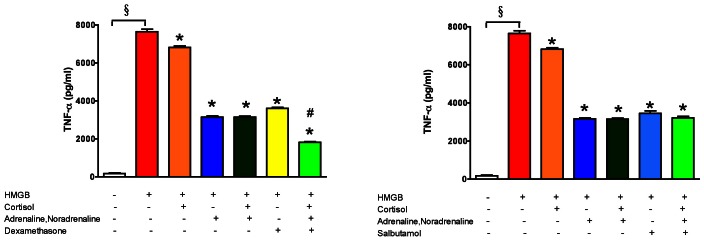
Inhibition of the HMGB-induced inflammatory response by endogenous catecholamines and glucocorticoids at physiological concentrations. RAW 264.7 cells were pretreated with cortisol (0.7 µM), noradrenaline (0.5 ng/ml), adrenaline (0.5 ng/ml), dexamethasone (1 µM) and salbutamol (1 µM) and exposed to HMGB1 (5 µg/ml) for 18 hours. TNFα secretion was measured in the supernatant. (^§^p<0.05 HMGB1-treated group compared to vehicle treated control, *p<0.05 compared to HMGB1 group, ^#^p<0.05 cells treated with all compounds in combination versus treated with a combination of two.).

Given the fact that both glucocorticoids and beta-receptor agonists represent endogenous hormones of the sympathetic-adrenal-medullary axis, we have next evaluated whether cortisol and/or adrenaline/noradrenaline, at concentrations that are comparable to their endogenous plasma levels, affect HMGB1-induced TNFα production. Cortisol, and, more markedly, the combination of adrenaline and noradrenaline, suppressed the HMGB1-induced TNFα response (Fig. 11).

### Glucocorticoid/beta-adrenergic Agonist Synergy *in vivo*

**Figure 12:**
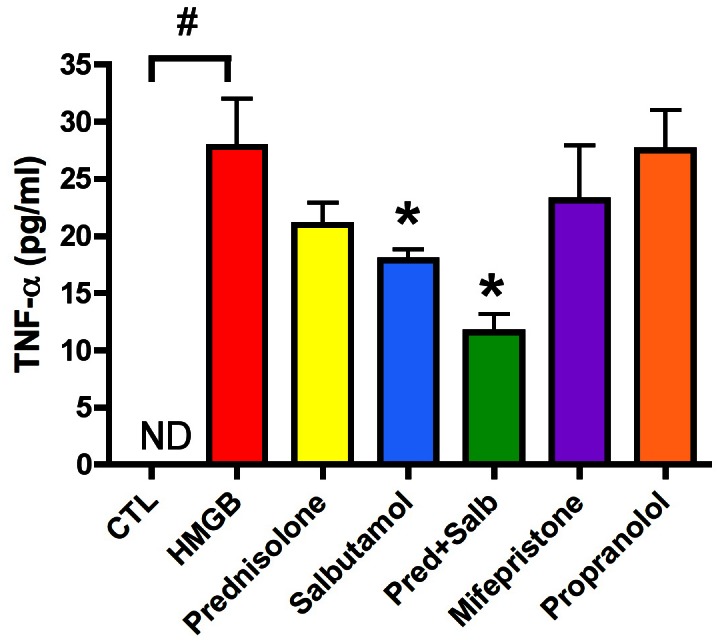
Inhibition of the HMGB-induced TNFα production by catecholamines and glucocorticoids *in vivo*. Balb/c male mice (Charles River Laboratories) were injected with 0.5 mg/kg HMGB1 in the presence of 60 min pretreatment of either vehicle, or 20 mg/kg prednisolone, 10 mg/kg salbutamol, the combination of prednisolone and salbutamol (doses as above), or the glucocorticoid receptor blocker mifepristone (30 mg/kg) or the β-receptor antagonist propranolol (10 mg/kg). At 8 hours after HMGB1 injection, animals were sacrificed and serum levels of TNFα were measured. ^#^p<0.05 represents a significant increase in TNFα serum levels in response to HMGB1; *p<0.05 represents significant inhibition of HMGB1-induced TNFα production by the various pharmacological agents indicated. n = 7 animals per group.

The combination of prednisolone and salbutamol effectively suppressed HMGB1-induced TNFα production in Balb/c male mice *in vivo*, and this effect was more pronounced than the effect of either agent alone (Fig. 12). In contrast, the glucocorticoid receptor blocker mifepristone or the β-receptor antagonist propranolol did not enhance HMGB1-induced TNFα production (Fig. 12), suggesting that the response is not under significant control by endogenous glucocorticoids acting on the mifepristone-sensitive glucocorticoid receptor or by endogenous catecholamines acting on the β-receptor.

## Discussion

It is well established that HMGB1 plays a central role in sterile inflammation [1]–[3]. This screen was undertaken to identify inhibitors of HMGB1-induced, TLR4 dependent TNFα production. The hit compounds emerging from the primary screen included several signal transduction pathway modifiers, such as the IκB phosphorylation inhibitor Bay 11-7085 and the Src/Syk kinase inhibitor MNS. These findings are consistent with a role of NF-κB and kinase activation in HMGB1-mediated cellular signaling. Glucocorticoids and beta-receptor agonist activators of intracellular cAMP (such as such as salbutamol, clenbuterol, metaproterenol, ethylnorepinephrine and ritodrine) were two most prominent drug classes emerging from the screen. Because of their therapeutic potential, and because of the endogenous physiological regulatory implications, these two classes of compounds were subject of follow-up studies (see below). Additional classes of hit compounds included the natural compounds, piperlongumine and parthenolide (the latter compound is known pharmacological actions as a NF-κB and MAP kinase inhibitor). The mechanism of action and potential anti-inflammatory utility of miscellaneous additional compounds that showed inhibitory effects - such as the HIV protease inhibitor indinavir, the local anesthetic lidocaine, the surfactant tyloxapol, the calcium antagonist prenylamine and the diuretic ethacrynic acid - requires further characterization. It is interesting to note that indinavir [18], tyloxapol [19] and lidocaine [20] have previously been demonstrated to suppress TNFα production in various experimental models *in vitro*, although the underlying molecular pathways have not been fully characterized. It is intriguing to speculate that these compounds may have additional, hitherto unrecognized, secondary modes of pharmacological action (as well as potential therapeutic utility) due to inhibition of HMGB1-mediated inflammatory responses.

We identified several different activators of intracellular cAMP signaling as part of the screen for enhancers of the inhibitory effect of glucocorticoids. The enhancers exerted their effects their effects either through beta-adrenergic receptor agonism (such as salbutamol and salmeterol), through prolongation of the intracellular half-life of cAMP (such as the phosphodiesterase inhibitor rolipram) or by activating the cAMP-dependent protein kinase (PKA) (such as prostaglandin E1). While neither the glucocorticoids nor the cAMP-stimulating agents, on their own, produced a complete inhibition of HMGB1-mediated TNFα response, the combination of these two agents yielded a robust inhibition, and did so at low micromolar/nanomolar concentrations. Previous studies have demonstrated synergistic interactions between steroids and beta-agonists in various experimental systems *in vitro* and suggested that cAMP and glucocorticoids act via distinct upstream pathways, which activate transcription though separate hormone response elements, the glucocorticoid receptor (GR) element (GRE) and the cAMP-response element (CRE), respectively. The site of the synergistic convergence was identified at the level of inhibition of the promoter activation of various pro-inflammatory genes [21]–[23]. Based on our findings, at least some of the synergistic inhibition of HMGB1-induced signaling by the glucocorticoid/beta-agonist combination occurs upstream from NF-κB activation, upstream from GRE and CRE and upstream from the promoter region of the inflammatory genes studied.

Our analysis of the gene expression profiles using a TLR signaling pathway array demonstrated that the synergistic inhibition of HMGB1-induced TNFα production by the glucocorticoid and the beta agonist does not represent a generalized phenomenon. In the case of several mediators, neither the glucocorticoid tested (e.g. Ccl2, Tlr2, Tlr9, Cd14, Cebpb, Csf3, Tlr8), nor the beta agonist tested (e.g. Il-1a, IL1b, IL6, Csf3, IL1r1) showed any inhibition. In some cases an enhancement was seen (IL1ra, Ptgs2, IL-10). These findings clearly demonstrate that HMGB1-mediated pro-inflammatory mediator production is regulated by glucocorticoids and by cAMP in a fashion that is specific to each gene product, and may be, at least in part, related to individual differences in the steroid and cAMP-responsive elements in individual promoters. Nevertheless, the combination of the beta agonist and the glucocorticoid resulted in a partial suppression for the majority of the genes studied, yielding a shift towards an overall anti-inflammatory phenotype (without suppressing the expression of the anti-inflammatory cytokine IL-10).

HMGB1 signals through numerous receptors, depending upon the molecular conformation of the three cysteines [2], [12]–[14], [24]–[26]. For example, extracellular HMGB1 is post-translationally regulated via redox mechanisms, and the C23-C45 disulfide C106 thiol conformation binds to and activates the TLR4/MD2 receptor complex in the absence of LPS [12]–[14]. Here we utilized this recombinant conformation of HMGB1 (purified and characterized as previously described), which primarily signals through TLR4 to induce TNFα. Because RAGE and TLR2 are dispensable for this effect, our studies would not be expected to address signaling mediated through these receptors.

Hormones of the hypothalamic-pituitary-adrenal axis, the sympathetic-adrenal-medullary axis, and the sympathetic and parasympathetic arms of the autonomic nervous system have powerful roles in the control of inflammation [27]–[31]. Adrenalectomy or pharmacological blockade of endogenous glucocorticoid receptors exacerbates [30], while beta-receptor activation suppresses systemic inflammatory responses [31]. Considering the fact that the biologically active concentrations of glucocorticoids and catecholamines in the current study are in the physiological range, we have also explored whether the HMGB1-mediated inflammatory responses are under the tonic control of these hormones. While the combination of exogenous glucocorticoid and beta agonist inhibited HMGB1-induced TNFα production (thereby extending the *in vitro* findings to an *in vivo* system), blockade of the endogenous glucocorticoid receptors with mifepristone or inhibition of the beta receptors with propranolol failed to potentiate the HMGB1-induced TNFα responses *in vivo*. Thus, circulating HMGB1 does not result in a more severe inflammatory response in subjects with impairment of their endogenous sympathetic-adrenal-medullary homeostasis, at least in the current *in vivo* experimental system.

In summary, the current study unveils several drug-like modulators of HMGB1-mediated inflammatory responses and offers pharmacological directions for the therapeutic suppression of inflammatory responses in diseases driven by the HMGB1-TLR4 axis. Glucocorticoids remain a mainstay of therapy for rheumatoid arthritis, as well as many other inflammatory diseases. In rheumatoid arthritis HMGB1 has been shown to play a significant pathogenetic role [2], [3]. We hypothesize that the mode of the therapeutic action of glucocorticoids, in addition to inhibiting HMGB1 release [11], also involves an inhibition of HMGB1's downstream signaling action. Furthermore, we conclude that the synergistic administration of a glucocorticoid and a beta-receptor agonist or (another cAMP-elevating agent) is an effective approach to suppress HMGB1-mediated inflammatory responses *in vitro* and *in vivo*.

## Supporting Information

Table S1 **A full list of the genes investigated in the real-time PCR array experiments.**
(XLS)

Table S2 **A full list of the primary data produced by the primary cell-based screens.**
(XLS)

## References

[1] Klune JR, Dhupar R, Cardinal J, Billiar TR, Tsung A (2008) HMGB1: endogenous danger signaling. Mol Med 14: 476–84. 18431461 10.2119/2008-00034.Klune PMC2323334

[2] Harris HE, Andersson U, Pisetsky D (2012) HMGB1: a multifunctional alarmin driving autoimmune and inflammatory disease. Nat Rev Rheumatol 8: 195–202. 22293756 10.1038/nrrheum.2011.222

[3] Andersson U, Tracey KJ (2011) HMGB1 is a therapeutic target for sterile inflammation and infection. Annu Rev Immunol 29: 139–62. 21219181 10.1146/annurev-immunol-030409-101323 PMC4536551

[4] Evankovich J, Cho SW, Zhang R, Cardinal J, Dhupar R, et al (2010) High mobility group box 1 release from hepatocytes during ischemia and reperfusion injury is mediated by decreased histone deacetylase activity. J Biol Chem 285: 39888–97. 20937823 10.1074/jbc.M110.128348 PMC3000970

[5] Lu B, Nakamura T, Inouye K, Li J, Tang Y, et al (2012) Novel role of PKR in inflammasome activation and HMGB1 release. Nature 488: 670–4. 22801494 10.1038/nature11290 PMC4163918

[6] Wang H, Ward MF, Sama AE (2009) Novel HMGB1-inhibiting therapeutic agents for experimental sepsis. Shock 32: 348–57. 19333143 10.1097/SHK.0b013e3181a551bd PMC2860725

[7] Yang H, Tracey KJ (2010) Targeting HMGB1 in inflammation. Biochim Biophys Acta 1799: 149–56. 19948257 10.1016/j.bbagrm.2009.11.019 PMC4533842

[8] Ulloa L, Ochani M, Yang H, Tanovic M, Halperin D, et al (2002) Ethyl pyruvate prevents lethality in mice with established lethal sepsis and systemic inflammation. Proc Natl Acad Sci USA 99: 12351–6. 12209006 10.1073/pnas.192222999 PMC129448

[9] Ditsworth D, Zong WX, Thompson CB (2007) Activation of poly(ADP)-ribose polymerase (PARP-1) induces release of the pro-inflammatory mediator HMGB1 from the nucleus. J Biol Chem 282: 17845–54. 17430886 10.1074/jbc.M701465200 PMC3140953

[10] Cai B, Chen F, Ji Y, Kiss L, de Jonge WJ, et al (2009) Alpha7 cholinergic-agonist prevents systemic inflammation and improves survival during resuscitation. J Cell Mol Med 13: 3774–85. 19602049 10.1111/j.1582-4934.2008.00550.x PMC3046874

[11] Schierbeck H, Wähämaa H, Andersson U, Harris HE (2010) Immunomodulatory drugs regulate HMGB1 release from activated human monocytes. Mol Med 16: 343–51. 20386869 10.2119/molmed.2010.00031 PMC2935946

[12] Venereau E, Casalgrandi M, Schiraldi M, Antoine DJ, Cattaneo A, et al (2012) Mutually exclusive redox forms of HMGB1 promote cell recruitment or proinflammatory cytokine release. J Exp Med. 209: 1519–28. 10.1084/jem.20120189 PMC3428943 22869893

[13] Yang H, Lundbäck P, Ottosson L, Erlandsson-Harris H, Venereau E, et al (2012) Redox modification of cysteine residues regulates the cytokine activity of high mobility group box-1 (HMGB1). Mol Med. 18: 250–9. 10.2119/molmed.2011.00389 PMC3324950 22105604

[14] Kim S, Kim SY, Pribis JP, Lotze M, Mollen KP, et al.. (2013) Signaling of High Mobility Group Box 1 (HMGB1) through toll-like receptor 4 in macrophages requires CD14. Mol Med, in press. 10.2119/molmed.2012.00306 PMC3667211 23508573

[15] Yang H, Hreggvidsdottir HS, Palmblad K, Wang H, Ochani M, et al (2010) A critical cysteine is required for HMGB1 binding to Toll-like receptor 4 and activation of macrophage cytokine release. Proc Natl Acad Sci USA. 107: 11942–11947. 10.1073/pnas.1003893107 PMC2900689 20547845

[16] Gero D, Modis K, Nagy N, Szoleczky P, Toth ZD, et al (2007) Oxidant-induced cardiomyocyte injury: identification of the cytoprotective effect of a dopamine 1 receptor agonist using a cell-based high-throughput assay. Int J Mol Med 20: 749–61. 17912470

[17] Gero D, Szoleczky P, Suzuki K, Modis K, Olah G, et al (2013) Cell-based screening identifies paroxetine as an inhibitor of diabetic endothelial dysfunction. Diabetes 62: 953–64. 23223176 10.2337/db12-0789 PMC3581231

[18] Lagathu C, Bastard JP, Auclair M, Maachi M, Kornprobst M, et al (2004) Antiretroviral drugs with adverse effects on adipocyte lipid metabolism and survival alter the expression and secretion of proinflammatory cytokines and adiponectin in vitro. Antivir Ther 9: 911–20. 15651750

[19] Ghio AJ, Marshall BC, Diaz JL, Hasegawa T, Samuelson W, et al (1996) Tyloxapol inhibits NF-κB and cytokine release, scavenges HOCI, and reduces viscosity of cystic fibrosis sputum. Am J Respir Crit Care Med 154: 783–8. 8810619 10.1164/ajrccm.154.3.8810619

[20] Lahat A, Ben-Horin S, Lang A, Fudim E, Picard O, et al (2008) Lidocaine down-regulates nuclear factor-κB signalling and inhibits cytokine production and T cell proliferation. Clin Exp Immunol 152: 320–7. 18355353 10.1111/j.1365-2249.2008.03636.x PMC2384107

[21] Park EA, Gurney AL, Nizielski SE, Hakimi P, Cao Z, et al (1993) Relative roles of CCAAT/Enhancer-binding protein beta and cAMP regulatory element-binding protein in controlling transcription of the gene for phosphoenolpyruvate carboxykinase. J Biol Chem 268: 613–619. 8093246

[22] Barnes PJ (2002) Scientific rationale for inhaled combination therapy with long-acting beta2-agonists and corticosteroids. Eur Respir J 19: 182–91. 11843317 10.1183/09031936.02.00283202

[23] Colangelo AM, Mallei A, Johnson PF, Mocchetti I (2004) Synergistic effect of dexamethasone and beta-adrenergic receptor agonists on the nerve growth factor gene transcription. Brain Res Mol Brain Res. 124: 97–104. 10.1016/j.molbrainres.2004.01.011 15135217

[24] Hori O, Brett J, Slattery T, Cao R, Zhang J, et al (1995) The receptor for advanced glycation end products (RAGE) is a cellular binding site for amphoterin. Mediation of neurite outgrowth and co-expression of rage and amphoterin in the developing nervous system. J Biol Chem 270: 25752–61. 7592757 10.1074/jbc.270.43.25752

[25] Chen GY, Tang J, Zheng P, Liu Y (2009) CD24 and Siglec-10 selectively repress tissue damage-induced immune responses. Science 323: 1722–1725. 19264983 10.1126/science.1168988 PMC2765686

[26] Bianchi ME (2009) HMGB1 loves company. J Leukoc Biol 86: 573–576. 19414536 10.1189/jlb.1008585

[27] Selye H (1971) Hormones and resistance. J Pharm Sci 60: 1–28. 4926765 10.1002/jps.2600600102

[28] Szelenyi J, Vizi ES (2007) The catecholamine cytokine balance: interaction between the brain and the immune system. Ann N Y Acad Sci 1113: 311–24. 17584982 10.1196/annals.1391.026

[29] Tracey KJ (2009) Reflex control of immunity. Nat Rev Immunol 9: 418–28. 19461672 10.1038/nri2566 PMC4535331

[30] Bertini R, Bianchi M, Ghezzi P (1988) Adrenalectomy sensitizes mice to the lethal effects of interleukin 1 and tumor necrosis factor. J Exp Med 167: 1708–12. 3259257 10.1084/jem.167.5.1708 PMC2188949

[31] Szabo C, Hasko G, Zingarelli B, Nemeth ZH, Salzman AL, et al (1997) Isoproterenol regulates tumour necrosis factor, interleukin-10, interleukin-6 and nitric oxide production and protects against the development of vascular hyporeactivity in endotoxaemia. Immunology 90: 95–100. 9038718 10.1046/j.1365-2567.1997.00137.x PMC1456713

